# Synthesis of M-Ag_3_PO_4_, (M = Se, Ag, Ta) Nanoparticles and Their Antibacterial and Cytotoxicity Study

**DOI:** 10.3390/ijms231911403

**Published:** 2022-09-27

**Authors:** Faiza Qureshi, Muhammad Nawaz, Mohammad Azam Ansari, Firdos Alam Khan, Mahmoud M. Berekaa, Samar A. Abubshait, Rayyanah Al-Mutairi, Alok K. Paul, Veeranoot Nissapatorn, Maria de Lourdes Pereira, Polrat Wilairatana

**Affiliations:** 1 Deanship of Scientific Research, Imam Abdulrahman Bin Faisal University, P.O. Box 1982, Dammam 31441, Saudi Arabia; 2 Department of Nano-Medicine Research, Institute for Research and Medical Consultations (IRMC), Imam Abdulrahman Bin Faisal University, P.O. Box 1982, Dammam 31441, Saudi Arabia; 3 Department of Epidemic Disease Research, Institutes for Research and Medical Consultations (IRMC), Imam Abdulrahman Bin Faisal University, P.O. Box 1982, Dammam 31441, Saudi Arabia; 4 Department of Stem Cell Research, Institute for Research and Medical Consultations (IRMC), Imam Abdulrahman Bin Faisal University, P.O. Box 1982, Dammam 31441, Saudi Arabia; 5 Environmental Health Department, College of Public Health, Imam Abdulrahman Bin Faisal University, P.O. Box 1982, Dammam 31441, Saudi Arabia; 6 Department of Chemistry, College of Science and Basic & Applied Scientific Research Centre, Imam Abdulrahman Bin Faisal University, P.O. Box 1982, Dammam 31441, Saudi Arabia; 7 School of Pharmacy and Pharmacology, University of Tasmania, Hobart, TAS 7001, Australia; 8 School of Allied Health Sciences, World Union for Herbal Drug Discovery (WUHeDD), and Research Excellence Center for Innovation and Health Products (RECIHP), Walailak University, Nakhon Si Thammarat 80160, Thailand; 9 CICECO-Aveiro Institute of Materials & Department of Medical Sciences, University of Aveiro, 3810-193 Aveiro, Portugal; 10 Department of Clinical Tropical Medicine, Faculty of Tropical Medicine, Mahidol University, Bangkok 10400, Thailand

**Keywords:** Ag_3_PO_4_, Se-Ag_3_PO_4_, Ag-Ag_3_PO_4_, Ta-Ag_3_PO_4_, nanoparticles, antibacterial, cytotoxicity

## Abstract

Silver Phosphate, Ag_3_PO_4_, being a highly capable clinical molecule, an ultrasonic method was employed to synthesize the M-Ag_3_PO_4,_ (M = Se, Ag, Ta) nanoparticles which were evaluated for antibacterial and cytotoxicity activities post-characterization. *Escherichia coli* and *Staphylococcus aureus* were used for antibacterial testing and the effects of sonication on bacterial growth with sub-MIC values of M-Ag_3_PO_4_ nanoparticles were examined. The effect of M-Ag_3_PO_4_ nanoparticles on human colorectal carcinoma cells (HCT-116) and human cervical carcinoma cells (HeLa cells) was examined by MTT (3-(4,5-dimethylthiazol-2-yl)-2,5-diphenyl-2H-tetrazolium bromide) assay and DAPI (4′,6-diamidino-2-phenylindole) staining. Additionally, we analyzed the effect of nanoparticles on normal and non-cancerous human embryonic kidney cells (HEK-293). Ag-Ag_3_PO_4_ exhibited enhanced antibacterial activity followed by Ta-Ag_3_PO_4,_ Ag_3_PO_4_, and Se-Ag_3_PO_4_ nanoparticles against *E. coli*. Whereas the order of antibacterial activity against *Staphylococcus aureus* was Ag_3_PO_4_ > Ag-Ag_3_PO_4_ > Ta-Ag_3_PO_4_ > Se-Ag_3_PO_4_, respectively. Percentage inhibition of *E. coli* was 98.27, 74.38, 100, and 94.2%, while percentage inhibition of *S. aureus* was 25.53, 80.28, 99.36, and 20.22% after treatment with Ag_3_PO_4_, Se-Ag_3_PO_4_, Ag-Ag_3_PO_4_, and Ta-Ag_3_PO_4,_ respectively. The MTT assay shows a significant decline in the cell viability after treating with M-Ag_3_PO_4_ nanoparticles. The IC_50_ values for Ag_3_PO_4,_ Se-Ag_3_PO_4,_ Ag-Ag_3_PO_4_, and Ta-Ag_3_PO_4_ on HCT-116 were 39.44, 28.33, 60.24, 58.34 µg/mL; whereas for HeLa cells, they were 65.25, 61.27, 75.52, 72.82 µg/mL, respectively. M-Ag_3_PO_4_ nanoparticles did not inhibit HEK-293 cells. Apoptotic assay revealed that the numbers of DAPI stained cells were significantly lower in the M-Ag_3_PO_4_-treated cells versus control.

## 1. Introduction

Nanotechnology is considered an advanced research field; nanoparticles with diverse shape, size, chemical properties, and different potential applications have been achieved [1,2,3]. Nanoparticles reveal several advantages over bulk material such as a large surface area, controlled shape, and size [4,5]. They are widely used in the diagnosis and treatment of diseases [6,7]. Due to their small size, several drugs can be delivered by using nanoparticles [8,9,10,11,12,13,14]. Different nanoparticles have been used as drug enhancers to improve the stability, efficacy, treatment, and safety of anti-cancer drugs [15,16,17,18]. 

Drug resistance is a worldwide issue and threat; many diseases caused by bacteria have a serious effect on public health. Although antibiotics influence bacteria, none of them is efficiently effective against multi-resistance bacteria [19,20,21]. Currently, some silver-based compounds such as silver nitrate, silver sulfadiazine, and silver alloy have been used to cure surgical incision, burns, ulcers, blood, and urinary infections [22]. Ag_3_PO_4_ (Silver orthophosphate) is a novel material and considered important due to its high photocatalytic activity under visible light irradiation. It is also effective at killing bacteria and fungi [23,24,25] and has even higher activity than streptomycin [26]. The biological activity is enhanced in conjugation [27,28,29]. Zhuang et al. [30] tested Ag_3_PO_4_/AgBr for enhanced anticorrosion photocatalysis. Gao et al. [31] added nano Ag with Ag_3_PO_4_ as a stable photocatalyst under visible light. Xiaohong et al. [32] prepared a powdered film Ag_3_PO_4_@AgBr and tested antibacterial activity; they exhibited a broad spectrum against *Escherichia coli* (*E. coli*) and *Staphylococcus aureus* (*S. aureus*). Similarly, Hossein et al. [33] reported Ag_3_PO_4_/GO membrane and evaluated antibacterial activity against *S. aureus* and *E. coli* and that the reduction in colonies was 72–84%. Another group, Kaili et al. [34], demonstrated that ZnO/Ag_3_PO_4_ revealed enhanced antibacterial activity against *E. coli* and *S. aureus.* Qinqing et al. [35] observed that Bi_2_MoO_6_/Ag_3_PO_4_ exhibited good antibacterial activity against *E. coli* and *S. aureus* and that an increased concentration of silver resulted in higher antibacterial activity. In another study, Ying-hai [36] prepared an Ag_3_PO_4_/TiO_2_ heterostructure and noticed that Ag_3_PO_4_/TiO_2_ showed antibacterial activity against *E. coli* and *S. aureus*.

However, Ag_3_PO_4_ has less stability and undergoes photo-corrosion which limits its practical application [37,38]. It is necessary to add a probable sacrificial agent or enhanced and quick capture of photo-generated electrons during photocatalysis. Since photo-corrosion leads to a dissociation of Ag^+^ from the Ag_3_PO_4_ lattice, either combination of nano Ag [30] or the addition of an electron acceptor [31] such as selenium and tantalum, as nanocomposites may prevent it. Previously, Ag_3_PO_4-_based nanocomposites such as Ag_3_PO_4_@AgBr [32], Ag_3_PO_4_/GO [33], ZnO/Ag_3_PO_4_ [34], Bi_2_MoO_6_/Ag_3_PO_4_ [35], and Ag_3_PO_4_/TiO_2_ [36] were investigated for their photocatalytic and antibacterial activities.

Selenium, a vital micronutrient, in nano size exhibited anti-cancer, anti-inflammatory and antimicrobial potency, alone or in conjugation with other therapeutic agents, without any toxicity [39,40]. Tantalum reported with no inherent antimicrobial properties but was found to supplement the prevention of infection and microbial growth owing to its surface properties [41]. It is intriguing to use selenium and tantalum together with Ag_3_PO_4_ against microbes and cancer cells. 

Many synthetic approaches have been used by researchers for the nano preparation of silver therapeutic agents, such as bioreduction [42,43], green synthesis [44,45], electrospinning [21], precipitation [21], etc. Herein, we report a simple ultrasonic method for the preparation of Ag_3_PO_4_, Se-Ag_3_PO_4_, Ag-Ag_3_PO_4_, and Ta-Ag_3_PO_4_ nanoparticles. The crystal phases, size, and morphologies were analyzed. The antibacterial investigations were made against both Gram-positive *S. aureus* and Gram-negative *E. coli*. The cytotoxicity of Ag_3_PO_4_, Se-Ag_3_PO_4_, Ag-Ag_3_PO_4_, and Ta-Ag_3_PO_4_ nanoparticles was studied against HCT-116 and HeLa cells (human colorectal carcinoma & cervical carcinoma cells) and healthy HEK-293 (embryonic kidney cells).

## 2. Results and Discussion

### 2.1. Characterization of Ag_3_PO_4_, Se-Ag_3_PO_4,_ Ag-Ag_3_PO_4_, and Ta-Ag_3_PO_4_ Nanoparticles

**Figure 1:**
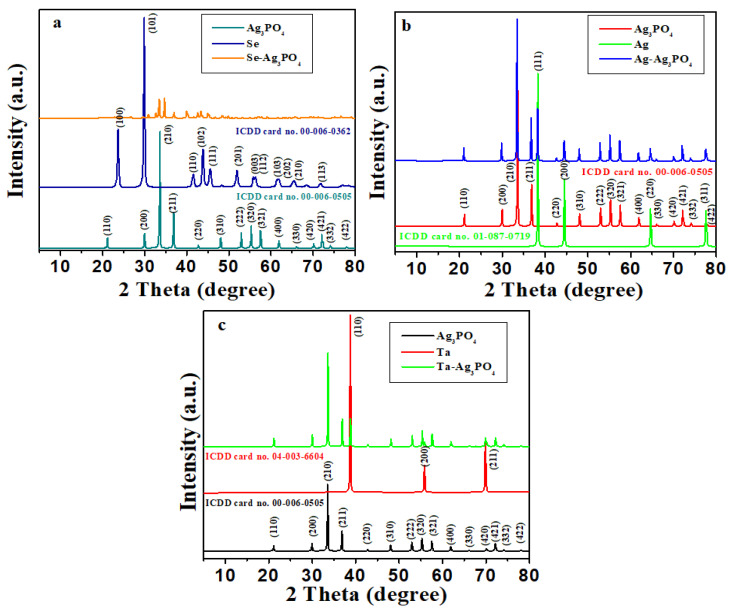
XRD pattern of Se-Ag_3_PO_4_ (**a**), Ag-Ag_3_PO_4_ (**b**), and Ta-Ag_3_PO_4_ (**c**).

**Figure 2:**
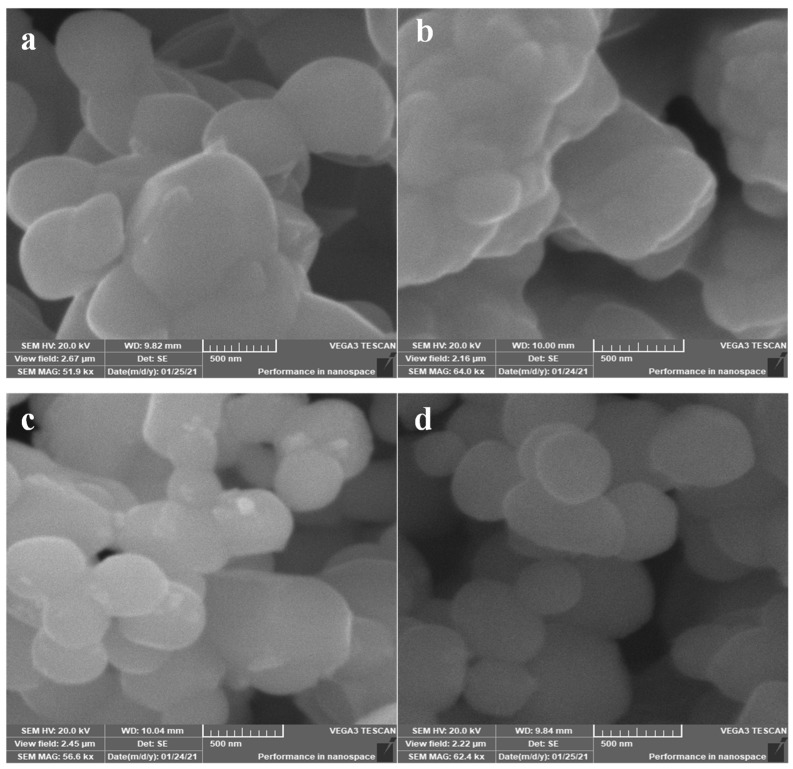
SEM images of Ag_3_PO_4_ (**a**), Se-Ag_3_PO_4_ (**b**), Ag-Ag_3_PO_4_ (**c**), and Ta-Ag_3_PO_4_ (**d**).

The XRD pattern of Ag_3_PO_4_, Se-Ag_3_PO_4,_ Ag-Ag_3_PO_4_, and Ta-Ag_3_PO_4_ nanoparticles is presented in Figure 1a–c. It has been observed that in all cases, peaks are well indexed with standard cards, confirming the formation of Se-Ag_3_PO_4,_ Ag-Ag_3_PO_4_, and Ta-Ag_3_PO_4_ nanoparticles. The peaks in Ag_3_PO_4_ correlate well with Ag_3_PO_4_ ICDD card no. 00-006-0505, showing the cubic structure. Se-Ag_3_PO_4,_ Ag-Ag_3_PO_4_, and Ta-Ag_3_PO_4_ nanoparticles exhibit related diffraction peaks similar to those of Ag_3_PO_4._ Similarly, Se, Ag, and Ta diffraction peaks correlate with ICDD card no. 00-006-0362, 01-087-0719, 04-003-6604, corresponding to hexagonal and cubic structures, respectively. The diffraction peaks of Se, Ag, and Ta matched with the Se-Ag_3_PO_4,_ Ag-Ag_3_PO_4_, and Ta-Ag_3_PO_4_ peaks. It was further observed that Ag-Ag_3_PO_4_ and Ta-Ag_3_PO_4_ exhibited the highest purity as compared with Se-Ag_3_PO_4_ nanoparticles. The morphology and size of Ag_3_PO_4_, Se-Ag_3_PO_4,_ Ag-Ag_3_PO_4_, and Ta-Ag_3_PO_4_ nanoparticles were investigated by SEM. The analysis of Figure 2a,b shows the formation of plate-like structure in the case of Ag_3_PO_4_ and Se-Ag_3_PO_4_ with an average size of 300–500 nm. However, Ag-Ag_3_PO_4_ and Ta-Ag_3_PO_4_ nanoparticles show the formation of nano-spheres with an average size of 300–500 nm (Figure 2a–d). Moreover, EDX analysis reveals the presence of Se, Ag, P, O_,_ and Ta in Se-Ag_3_PO_4,_ Ag-Ag_3_PO_4_, and Ta-Ag_3_PO_4_ nanoparticles (Figures S1–S4). Additionally, EDX mapping was performed to establish the distribution of Se, Ag, P, O_,_ and Ta in Se-Ag_3_PO_4,_ Ag-Ag_3_PO_4_, and Ta-Ag_3_PO_4_ nanoparticles. The results illustrate the successful preparation of Ag_3_PO_4_, Se-Ag_3_PO_4,_ Ag-Ag_3_PO_4_, and Ta-Ag_3_PO_4_ nanoparticles.

Zeta potential is a unique technique for determining the surface charge and stability of the nanoparticles. The zeta potential of Ag_3_PO_4_, Se-Ag_3_PO_4,_ Ag-Ag_3_PO_4_, and Ta-Ag_3_PO_4_ nanoparticles is presented in Figure S5. The zeta potential of Ag_3_PO_4_, Se-Ag_3_PO_4,_ Ag-Ag_3_PO_4_, and Ta-Ag_3_PO_4_ nanoparticles was observed as −40.1 ± 6.63, −5.24 ± 10.8, −46.6 ± 4.77, and −79.8 ± 7.96 mV, respectively. The zeta value greater than +30 mV or less than −30 mV indicated the stable colloidal dispersion. Our results revealed the high dispersion stability of Ta-Ag_3_PO_4_ nanoparticles followed by Ag-Ag_3_PO_4_, and Ag_3_PO_4_, while Se-Ag_3_PO_4_ nanoparticles indicated low stability. The particle size of Ag_3_PO_4_, Se-Ag_3_PO_4_, Ag-Ag_3_PO_4_, and Ta-Ag_3_PO_4_ nanoparticles was recorded as 115 (PDI: 0.509), 458 (PDI: 1.00), 426 (PDI: 0.949), and 82.78 nm (PDI: 0.594), respectively (Table 1). The results indicated that Se-Ag_3_PO_4,_ and Ag-Ag_3_PO_4_ nanoparticles have bigger particle size as compared to Ag_3_PO_4_, and Ta-Ag_3_PO_4_ nanoparticles. The polydispersity index (PDI) as well as the particle size of Ag_3_PO_4_ and Ta-Ag_3_PO_4_ was observed as lower, indicating their greater suitability for biomedical applications.

FTIR analysis was also performed to evaluate functional groups and the bonding of Ag_3_PO_4_, Se-Ag_3_PO_4,_ Ag-Ag_3_PO_4_, and Ta-Ag_3_PO_4_ nanoparticles. The peak at 544 cm^−1^ can be attributed to the P-O-P bending normal mode in Ag_3_PO_4_; another peak at 944 cm^−1^ represents the presence of P-O bonds [46]. Similarly, the P-O-P peak in Se-Ag_3_PO_4,_ Ag-Ag_3_PO_4_, and Ta-Ag_3_PO_4_ nanoparticles was observed at 838 cm^−1^, 946 cm^−1^, and 946 cm^−1^, respectively. Whereas P-O bonds peak in Se-Ag_3_PO_4,_ Ag-Ag_3_PO_4_, and Ta-Ag_3_PO_4_ nanoparticles was seen at 646 cm^−1^, 546 cm^−1^, and 547 cm^−1^, respectively (Figure S6a).

BET analysis of Ag_3_PO_4_, Se-Ag_3_PO_4,_ Ag-Ag_3_PO_4_, and Ta-Ag_3_PO_4_ nanoparticles was achieved to record the porosity and surface area. N_2_ adsorption/desorption isotherms are presented in Figure S6b, where Ag_3_PO_4_ and Se-Ag_3_PO_4_ do not show adsorption-desorption which could be due to the presence of the less porous structure of Ag_3_PO_4_ and Se-Ag_3_PO_4_. However, Ag-Ag_3_PO_4_ and Ta-Ag_3_PO_4_ nanoparticles exhibited N_2_-adsorption–desorption. The surface area of Ag_3_PO_4,_ Se-Ag_3_PO_4,_ Ag-Ag_3_PO_4_, and Ta-Ag_3_PO_4_ nanoparticles was 2.69 m^2^/g, 2.20 m^2^/g, 3.48 m^2^/g, and 2.61 m^2^/g respectively. While the pore size of Ag_3_PO_4,_ Se-Ag_3_PO_4,_ Ag-Ag_3_PO_4_, and Ta-Ag_3_PO_4_ nanoparticles was 2.15 nm, 1.93 nm, 2.83 nm, and 3.44 nm, respectively. Additionally, the pore volume of Ag_3_PO_4,_ Se-Ag_3_PO_4,_ Ag-Ag_3_PO_4_, and Ta-Ag_3_PO_4_ nanoparticles was 0.000605 cm^3^/g, 0.00047 cm^3^/g, 0.00128 cm^3^/g, and 0.00075 cm^3^/g, respectively. Since the pore size of the nanoparticles is less than 5 nm, it indicated the presence of micropores and mesopores [47]. The pore size of Ag_3_PO_4_, Se-Ag_3_PO_4_, Ag-Ag_3_PO_4_, and Ta-Ag_3_PO_4_ nanoparticles was 2.15 nm, 1.93 nm, 2.83 nm, and 3.44 nm respectively. After testing these nanoparticles on cancer cells, we found that cell viability significantly decreased after the treatments with Ag_3_PO_4_, Se-Ag_3_PO_4_, Ag-Ag_3_PO_4_, and Ta-Ag_3_PO_4_. 

DR-UV spectra of Ag_3_PO_4_, Se-Ag_3_PO_4,_ Ag-Ag_3_PO_4_, and Ta-Ag_3_PO_4_ nanoparticles were noted in the range 200–800 nm. All nanoparticles exhibited spectra in the visible range; however, in case of Se-Ag_3_PO_4_, wide spectra were observed with low absorption which could be due to the scattering of light in the pore structure of Se-Ag_3_PO_4_ (Figure S6c).

### 2.2. Antibacterial Activity Ag_3_PO_4_, Se-Ag_3_PO_4_, Ag-Ag_3_PO_4_, and Ta-Ag_3_PO_4_ Nanoparticles

**Figure 3:**
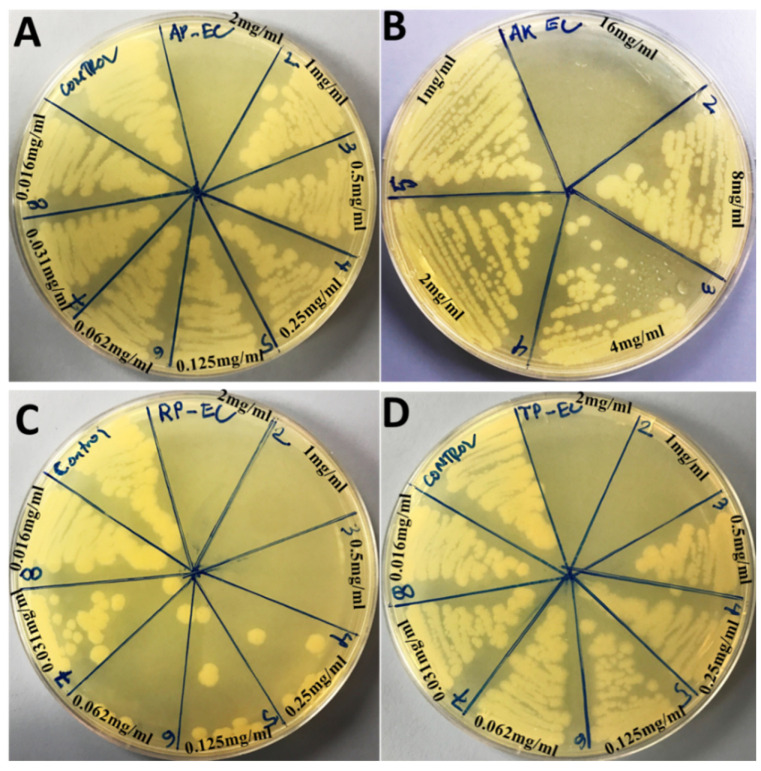
MHA plates showing MBC values for *E. coli* ATCC 25922. Plate (**A**) showing MBC value of 2 mg/mL for Ag_3_PO_4_, (**B**) 16 mg/mL for Se-Ag_3_PO_4_, (**C**) 0.5 mg/mL for Ag-Ag_3_PO_4_, and (**D**) 1 mg/mL for Ta-Ag_3_PO_4_, respectively.

**Figure 4:**
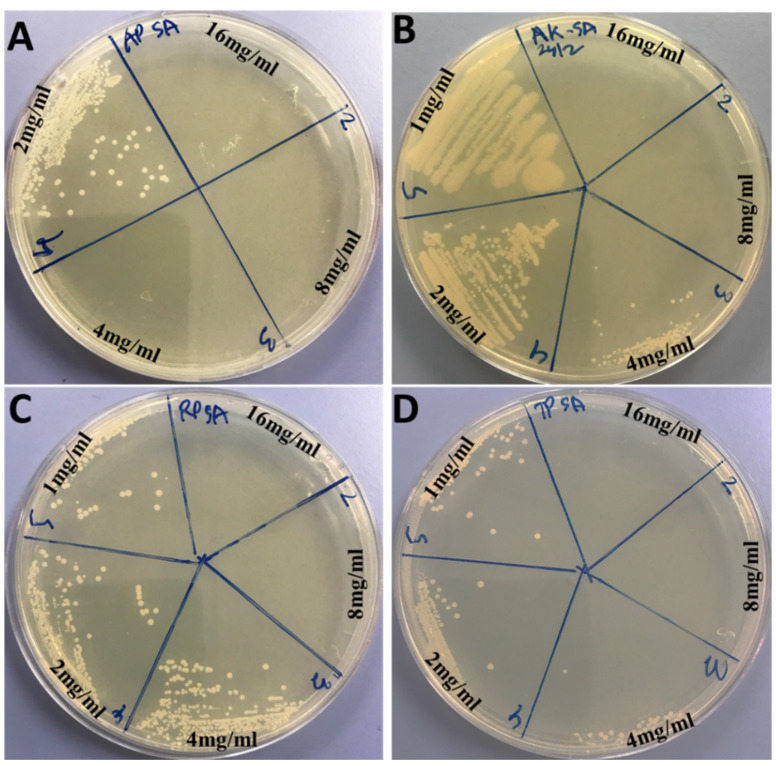
MHA plates showing MBC values for *S. aureus* ATCC 25923. Plate (**A**) showing MBC value of 4 mg/mL for Ag_3_PO_4_, (**B**) 8 mg/mL for Se-Ag_3_PO_4_, (**C**) 8 mg/mL for Ag-Ag_3_PO_4_, and (**D**) 8 mg/mL for Ta-Ag_3_PO_4_, respectively.

The antimicrobial activity of Ag_3_PO_4_, Se-Ag_3_PO_4_, Ag-Ag_3_PO_4_, and Ta-Ag_3_PO_4_ nanoparticles was examined against Gram-negative *E. coli* and Gram-positive *S. aureus* using a standard microbroth dilution method. The MICs and MBCs values of Ag_3_PO_4_, Se-Ag_3_PO_4_, Ag-Ag_3_PO_4_, and Ta-Ag_3_PO_4_ are represented in Table 2. It was observed that Ag-Ag_3_PO_4_ (MIC/MBC: 0.125/0.5 mg/mL) exhibited enhanced antibacterial activity followed by Ta-Ag_3_PO_4_ (MIC/MBC: 0.25/1 mg/mL)_,_ Ag_3_PO_4_ (MIC/MBC: 1/2 mg/mL), and Se-Ag_3_PO_4_ (MIC/MBC: 8/16 mg/mL) against *E. coli* (Table 2 and Figure 3). Whereas the order of antibacterial activity against *S. aureus* was as follows: Ag_3_PO_4_ (MIC/MBC: 2/4 mg/mL) > Ag-Ag_3_PO_4_ (MIC/MBC: 2/8 mg/mL) > Ta-Ag_3_PO_4_ (MIC/MBC: 4/8 mg/mL) > Se-Ag_3_PO_4_ (MIC/MBC: 4/8 mg/mL), respectively (Table 2 and Figure 4)_._ Small nanoparticle size possibly internalized bacterial cells, through ion diffusion and free radicals generation, which further enter the cells, destroying cellular components such as proteins, DNA, and lipids, as suggested by previous reports [48,49] that the antimicrobial activity increased due to a decrease in the particle size of nanoparticles. According to the findings of the MIC and MBC tests, it was found that Gram-negative bacteria, *E. coli*, were more susceptible to the tested nanoparticles than Gram-positive bacteria (*S. aureus*). The fact that the cell walls of these two species of bacteria are constructed differently may provide an explanation for this disparity. It is generally known that the principal component of the cell wall of Gram-positive bacteria is thick and rigid peptidoglycans (20–80 nm) that provide extra protection. In contrast, the cell wall of Gram-negative bacteria contains a thin layer of peptidoglycan (7–8 nm) and a highly negatively charged lipopolysaccharides layer, which may facilitate enhanced binding with the nanocomposite and result in more effective cell damage than Gram-positive bacteria [50].

Both MIC and MBC values are statistically significantly different (*p* =< 0.001) whereas the overall significance level = 0.05

### 2.3. Effects of Compounds on Bacteria Growth after Application of Sonication

**Figure 5:**
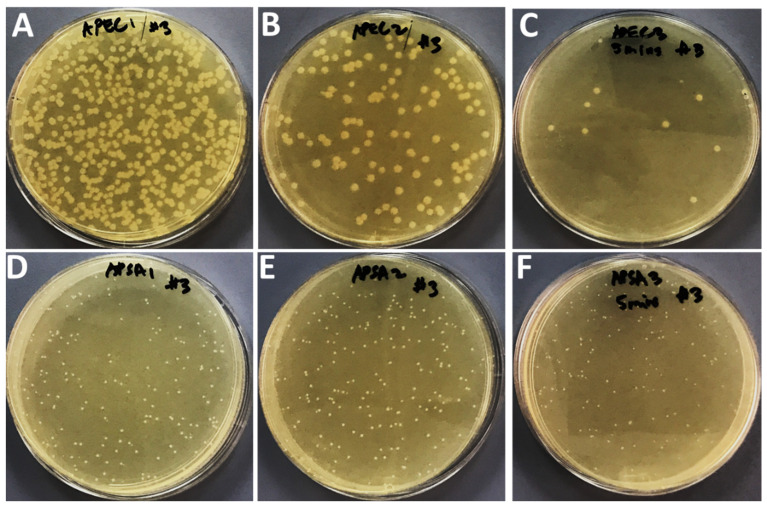
Effects of Ag_3_PO_4_ on the growth of *E. coli* (panel (**A**–**C**)) and *S. aureus* (panel (**D**–**F**)). (**A**,**D**); without compound and sonication, (**B**,**E**) with compound but without sonication, and (**C**,**F**); with compound and 5 min of sonication.

**Figure 6:**
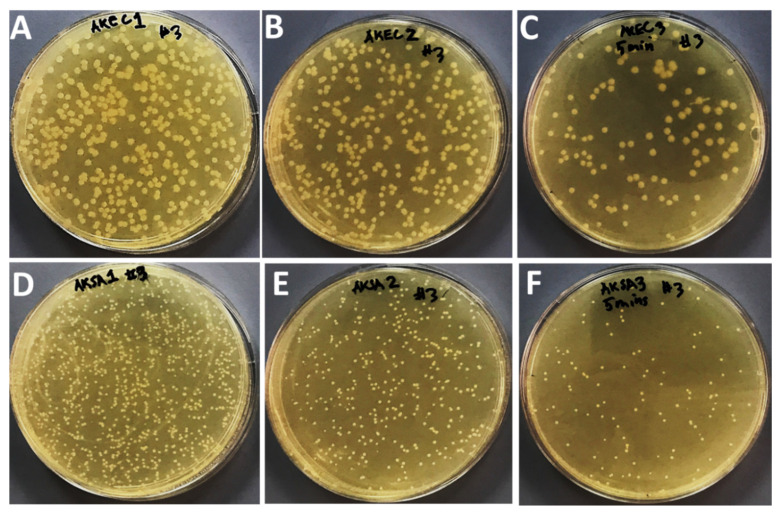
Effects of Se-Ag_3_PO_4_ on the growth of *E. coli* (**A**–**C**) and *S. aureus* (**D**–**F**). (**A**,**D**); without compound and without sonication, (**B**,**E**) with compound but without sonication, and (**C**,**F**); with compound and 5 min of sonication.

**Figure 7:**
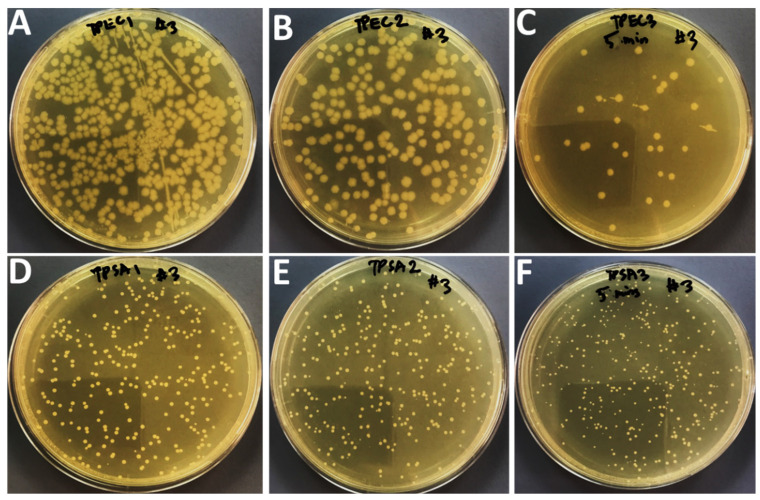
Effects of Ta-Ag_3_PO_4_ on the growth of *E. coli* (**A**–**C**) and S. *aureus* (**D**–F). (**A**,**D**); without compound and without sonication, (**B**,**E**) with compound but without sonication, and (**C**,**F**); with compound and 5 min of sonication.

**Figure 8:**
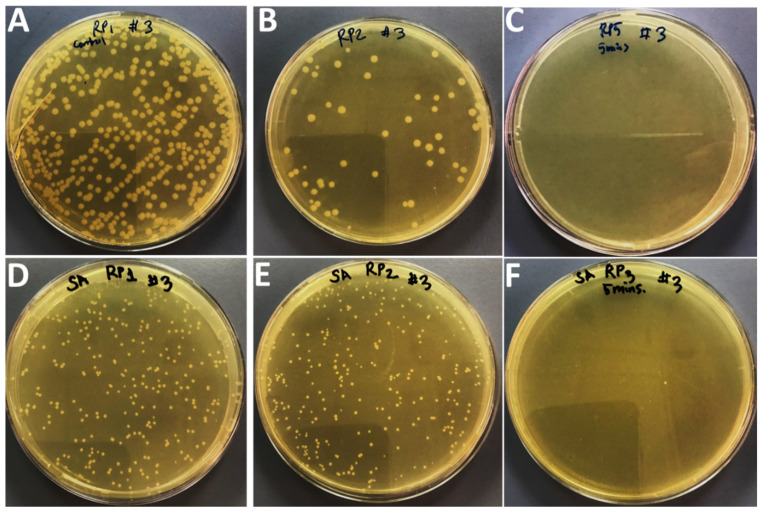
Effects of Ag-Ag_3_PO_4_ on the growth of *E. coli* (**A**–**C**) and *S. aureus* (**D**–**F**). (**A**,**D**); without compound and without sonication, (**B**,**E**) with compound but without sonication, and (**C**,**F**); with compound and 5 min of sonication.

**Figure 9:**
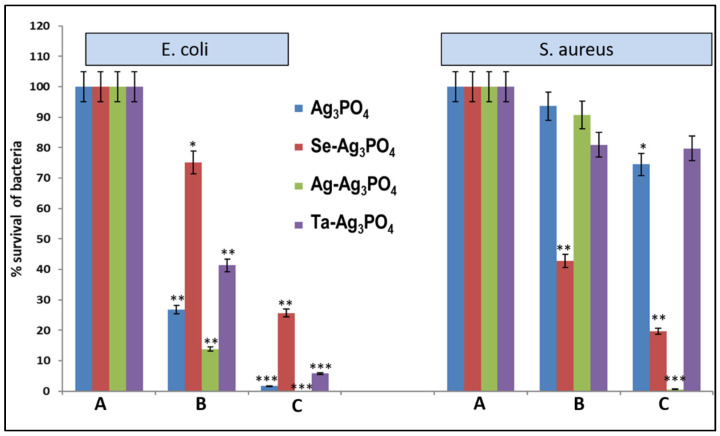
Effects of tested compounds on *E. coli* and *S. aureus* growth. A: control i.e., without nanoparticles and sonication; B: Treated with sub-MIC value of nanoparticle but without sonication; C: Treated with sub-MIC value of nanoparticle and sonication. * *p* < 0.05, ** *p* < 0.001; *** *p* < 0.0001.

The effects of treatment of sonication on bacterial growth in the presence of sub-MIC values of Ag_3_PO_4_, Se-Ag_3_PO_4_, Ag-Ag_3_PO_4_, and Ta-Ag_3_PO_4_ was also examined by the standard plate count method (Figure 5, Figure 6, Figure 7 and Figure 8) by calculating the percentage inhibition of bacterial growth cells (Figure 9). It was found that the viable cell count of bacteria cells was significantly reduced after 5 min of sonication treatment as compared to cells treated without the application of sonication (Figure 4, Figure 5, Figure 6 and Figure 7). It was observed that all the four compounds exhibit a pronounced effect on the survival of *E coli* and *S. aureus* after sonication. Furthermore, it was found that the percentage inhibition of *E. coli* was 98.27, 74.38, 100, and 94.2%, while % inhibition of *S. aureus* was 25.53, 80.28, 99.36, and 20.22% after treatment with Ag_3_PO_4_, Se-Ag_3_PO_4_, Ag-Ag_3_PO_4_, and Ta-Ag_3_PO_4_, respectively, after the application of sonication (Figure 7). It was found that, when compared to other tested compounds, the Ag-Ag_3_PO_4_ exhibits the highest antibacterial activity against both the tested bacterial strains. To the best of our knowledge, this is the first record where authors reported the impact of sonication on bacterial growth in the presence of Ag_3_PO_4_, Se-Ag_3_PO_4_, Ag-Ag_3_PO_4_, and Ta-Ag_3_PO_4_ nanoparticles. 

### 2.4. Effect of Ag_3_PO_4,_ Se-Ag_3_PO_4,_ Ag-Ag_3_PO_4,_ Ta-Ag_3_PO_4_ on Cancer Cells Viability

**Figure 10:**
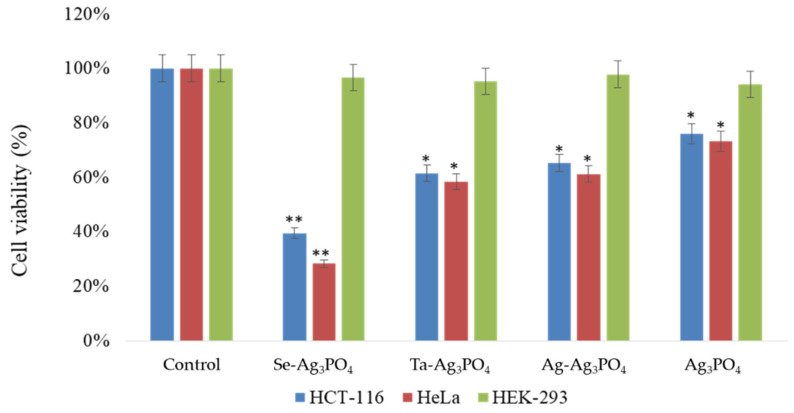
Cell viability using MTT Assay: It shows the impact of treatment of Ag_3_PO_4,_ Se-Ag_3_PO_4,_ Ag-Ag_3_PO_4,_ and Ta-Ag_3_PO_4_ on HCT-116 and HeLa cell viability post 48 h treatment. * *p* < 0.05; ** *p* < 0.01.

**Figure 11:**
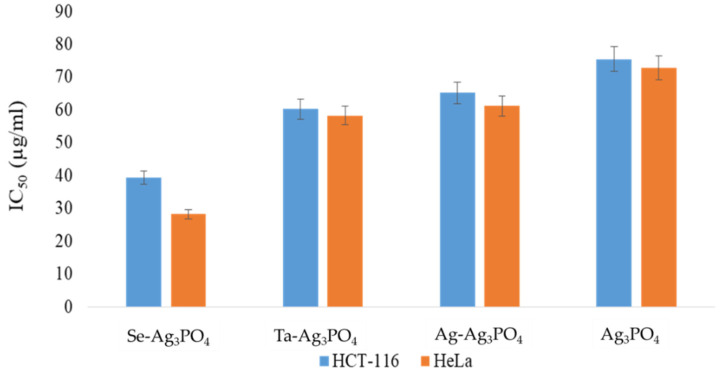
Average inhibitory concentration 50 (IC_50_) of Ag_3_PO_4,_ Se-Ag_3_PO_4,_ Ag-Ag_3_PO_4,_ Ta-Ag_3_PO_4_ on HCT-116 and HeLa cell. It shows the impact of treatment of Ag_3_PO_4,_ Se-Ag_3_PO_4,_ Ag-Ag_3_PO_4_, and Ta-Ag_3_PO_4_ on HCT-116 and HeLa cells post 48 h treatment.

The influence of Ag_3_PO_4,_ Se-Ag_3_PO_4,_ Ag-Ag_3_PO_4_, and Ta-Ag_3_PO_4_ on the two cell lines used in the study, colon carcinoma (HCT-116) and cervical cancer (HeLa), was investigated. The cell viability assay proved that cell viability significantly decreased after the treatments with Ag_3_PO_4,_ Se-Ag_3_PO_4,_ Ag-Ag_3_PO_4_, and Ta-Ag_3_PO_4_. The treatments Ag_3_PO_4,_ Se-Ag_3_PO_4,_ Ag-Ag_3_PO_4_, and Ta-Ag_3_PO_4_ indicated a dose-dependent inhibition of tumor cell growth and proliferation. HeLa cells showed better inhibitory action then HCT-116 cells (Figure 10). The impact of Ag_3_PO_4,_ Ag-Ag_3_PO_4_, and Ta-Ag_3_PO_4_ was also varied as Se-Ag_3_PO_4_ (pore size 1.93 nm) showed the greatest inhibitory action on both HeLa and HCT-116 cells, followed by Ag_3_PO_4_ (pore size 2.15 nm), Ag-Ag_3_PO_4_ (pore size 2.83 nm), and Ta-Ag_3_PO_4_ (pore size 3.44 nm) (Figure 11). Smaller nanoparticles showed more cytotoxicity on cancer cells than those with large pores. It has been shown in other studies that small nanoparticles produced better cytotoxic effects than large nanoparticles [51,52]. In one study, it was shown that polymeric NPs and poly(D,L-lactide-co-glycolide) (PLGA) NPs of 100 nm size demonstrated a more than threefold higher uptake compared to 275-nm size NPs in an ex-vivo canine carotid artery model [53]. In another study, it was found that gold nanoparticles with smaller diameters have superior membrane penetration than large-size gold nanoparticles [54].

The inhibitory concentration (IC_50_) of Ag_3_PO_4,_ Se-Ag_3_PO_4,_ Ag-Ag_3_PO_4,_ and Ta-Ag_3_PO_4_ was computed. The IC_50_ values for Ag_3_PO_4,_ Se-Ag_3_PO_4,_ Ag-Ag_3_PO_4,_ Ta-Ag_3_PO_4_ on HCT-116 cells were 39.44, 28.33, 60.24, 58.34 µg/mL; whereas for HeLa cells, they were 65.25, 61.27, 75.52, 72.82 µg/mL, respectively (Figure 11). 

The influence of Ag_3_PO_4,_ on HEK-293 cells was also analyzed and results showed that Ag_3_PO_4,_ Se-Ag_3_PO_4,_ Ag-Ag_3_PO_4,_ and Ta-Ag_3_PO_4_ did not have an inhibitory effect on HEK-293 cells. This suggests that prepared nanoparticles are safe for normal cells and do not cause any harm, whereas on cancer cells, the treatments induced significant cell death. While we do not know the molecular mechanism of the nanoparticles’ impact on normal cells, it has been shown that prepared nanoparticles are specifically targeted cells and induce cytotoxicity. This represents the first outcome demonstrating the cell viability of Ag_3_PO_4,_ Se-Ag_3_PO_4,_ Ag-Ag_3_PO_4_, and Ta-Ag_3_PO_4_ against HCT-116 and HeLa cells. Some researchers have published multiple reports on different molecules (nanomaterials and plant extracts) and their influence on colon and breast cancer cells [2,3,55,56,57,58,59].

### 2.5. Apoptotic Effect of Ag_3_PO_4,_ Se-Ag_3_PO_4,_ Ag-Ag_3_PO_4,_ Ta-Ag_3_PO_4_

**Figure 12:**
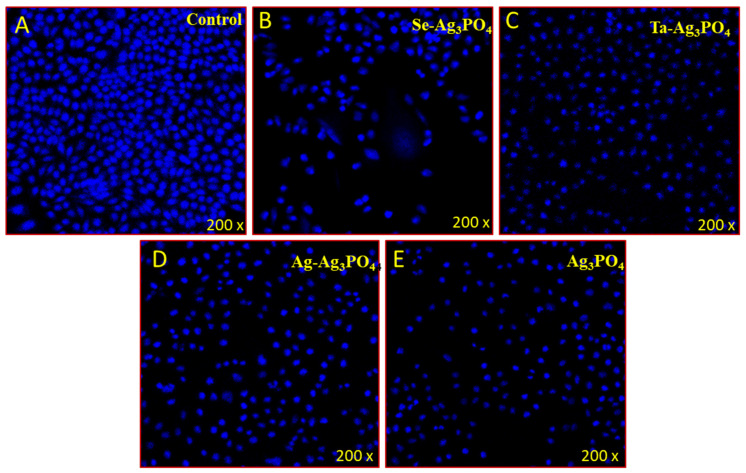
Cancer cell death due treatment of Ag_3_PO_4_, Se-Ag_3_PO_4_, Ag-Ag_3_PO_4_, and Ta-Ag_3_PO_4_. It shows the impact of the treatment of Ag_3_PO_4,_ Se-Ag_3_PO_4,_ Ag-Ag_3_PO_4_, and Ta-Ag_3_PO_4_ on HCT-116 cells stained with DAPI post 48-h treatment. (**A**) is the control cell and (**B**–**E**) are Ag_3_PO_4_, Se-Ag_3_PO_4,_ Ag-Ag_3_PO_4,_ Ta-Ag_3_PO_4_, where a significant number of death cancer cells are observed upon (40 µg/mL) treatment.

In the present study we used DAPI (4’,6-diamidino-2-phenylindole) to examine the cancer cell DNA after the treatments. DAPI is a fluorescent stain that binds strongly to AT-rich regions in the DNA. DAPI is a blue-fluorescent DNA stain that exhibits ~20-fold enhancement of fluorescence upon binding to AT regions of dsDNA. Because of its high affinity for DNA, it is also frequently used for counting cells, measuring apoptosis, sorting cells based on DNA content, and as a nuclear segmentation tool in high-content imaging analysis. The treatment of Ag_3_PO_4,_ Se-Ag_3_PO_4,_ Ag-Ag_3_PO_4_, and Ta-Ag_3_PO_4_ resulted in a significant decrease in the number of colon cancer cells, as the number of DAPI-stained cells appears to be substantially lower in the Ag_3_PO_4,_ Se-Ag_3_PO_4,_ Ag-Ag_3_PO_4,_ Ta-Ag_3_PO_4_-treated cells vs control cells (Figure 12B–D). The decline in cancer cells is the result of after the programmed cell death or apoptosis, whereas the control group did not show any inhibition towards colon cancer cells (Figure 12A). In addition, we also observed that Ag_3_PO_4_, Se-Ag_3_PO_4_, Ag-Ag_3_PO_4_, and Ta-Ag_3_PO_4_-treated cells showed change in the cancer nuclei morphology as they shrank (Figure 12B–E), compared to control cells (Figure 12A), which suggests that cancer cells are undergoing apoptosis.

## 3. Experimental

### 3.1. Materials and Methods

All materials and chemicals used in this study were purchased from commercial sources and used as commercial materials and chemicals. 

#### 3.1.1. Preparation of Ag_3_PO_4_, Se-Ag_3_PO_4_, Ag-Ag_3_PO_4_, and Ta-Ag_3_PO_4_ Nanoparticles

**Figure 13:**
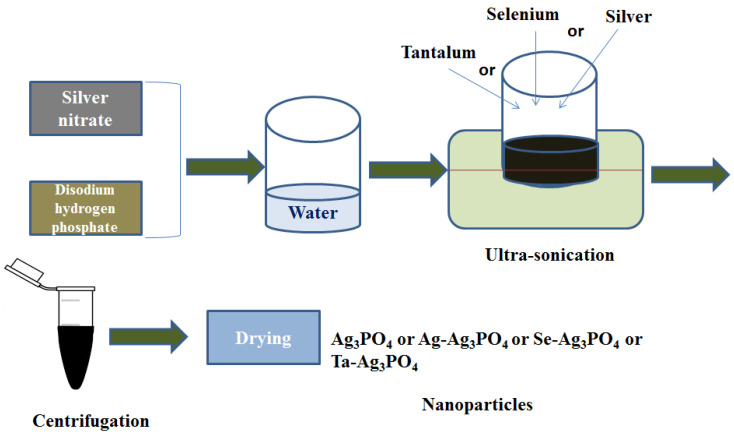
Schematic representation of Ag_3_PO_4_, Se-Ag_3_PO_4_, Ag-Ag_3_PO_4_, and Ta-Ag_3_PO_4_ nanoparticles.

0.5 g silver nitrate was added to 30 mL of water in a beaker. After sonicating for 5 min, 0.3 g disodium hydrogen phosphate (Na_2_HPO_4_) in 10 mL of water was added dropwise to the silver nitrate solution and ultra-sonicated for 20 min. After that, 0.2 g silver or selenium or tantalum powder was added to the ultra-sonication mixture and sonication was carried on for a further 40 min. The products were centrifuged, washed with water/ethanol and dried to give Se-Ag_3_PO_4_, Ag-Ag_3_PO_4_, and Ta-Ag_3_PO_4_. The same procedure was repeated to prepare Ag_3_PO_4_ except for the addition of silver or selenium, or tantalum powder (Figure 13).

#### 3.1.2. Characterization of Ag_3_PO_4_, Se-Ag_3_PO_4_, Ag-Ag_3_PO_4_, and Ta-Ag_3_PO_4_ Nanoparticles

X-ray diffraction (Rigaku, Japan) was performed to examine the phases of Ag_3_PO_4_, Se-Ag_3_PO_4_, Ag-Ag_3_PO_4_, and Ta-Ag_3_PO_4_ nanoparticles in the range of 10–80° with 0.9°/minute scanning speed. Scanning electron microscopic studies (SEM, Tscan,Brno-Kohoutovice, Czech Republic) of the as-synthesized Ag_3_PO_4_, Se-Ag_3_PO_4_, Ag-Ag_3_PO_4_, and Ta-Ag_3_PO_4_ nanoparticles were performed for the surface morphology and structure. Zeta size and zeta potential of the nanoparticles were determined by Malvern Zetasizer instrument, Malvern, United Kingdom (UK). Before analysis, samples were dispersed very well inthe deionized water by ultra-sonication. The diffuse reflectance of Ag_3_PO_4_, Se-Ag_3_PO_4_, Ag-Ag_3_PO_4_, and Ta-Ag_3_PO_4_ nanoparticles were measured using UV-visiblespectrophotometer (JASCO V-750,Helsinki, Finland) and FTIR spectra were recorded on a PerkinElmer spectrometer, Boston, Massachusetts, United States (USA). Micromeritics ASAP 2020 Plus (Norcross, USA) was used to analyze the surface area of Ag_3_PO_4_, Se-Ag_3_PO_4_, Ag-Ag_3_PO_4_, and Ta-Ag_3_PO_4_ nanoparticles withprior degassing for 2H at 180 °C.

#### 3.1.3. Antibacterial Activity of Ag_3_PO_4_, Se-Ag_3_PO_4_, Ag-Ag_3_PO_4_, and Ta-Ag_3_PO_4_ Nanoparticles

To evaluate the antibacterial activity of synthesized Ag_3_PO_4_, Se-Ag_3_PO_4_, Ag-Ag_3_PO_4_, and Ta-Ag_3_PO_4_, *E. coli* ATCC 25922 and *S. aureus* ATCC 25923 were used as model Gram -negative and Gram-positive. The bacteria were incubated overnight at 37 °C in a shaker incubator, and then harvested, and the biomass was washed using PBS to remove any remaining media before being used in the experiment.

#### 3.1.4. Minimal Inhibitory and Minimal Bactericidal Concentration (MIC & MBC) 

The minimum inhibitory concentration (MIC) potential of Ag_3_PO_4_, Se-Ag_3_PO_4_, Ag-Ag_3_PO_4_, and Ta-Ag_3_PO_4_ was investigated by standard microbroth dilution procedure in a 96-well round bottom microtiter plate. Briefly, 20 µL of freshly grown culture of each tested organism (0.5Macfarland) was inoculated in 180 µL of BHI broth containing a varying concentration (32–0.03125 mg/mL) of tested Ag_3_PO_4_, Se-Ag_3_PO_4_, Ag-Ag_3_PO_4_, and Ta-Ag_3_PO_4_ for 24h at 37 °C. MIC being the lowest concentration of antimicrobial agents which visually inhibit 99% growth of bacteria. The minimum bactericidal concentration (MBC) potential of tested materials was performed on the MHA plates. MBC is defined as the lowest concentration of tested compounds which kill 99.99% of the bacteria population. For the MBC test, 100 μL suspensions from each well of microtitre plates was spread onto the MHA plates and further incubated for 24 h at 37 °C. The lowest concentration with no visible growths on the MHA plate was considered as the MBC value [60]. 

#### 3.1.5. Synergistic Effects of Nanocomposites and Sonication on Bacteria Growth

Standard plate count procedures were used to further investigate the effects of Ag_3_PO_4_, Se-Ag_3_PO_4_, Ag-Ag_3_PO_4_, and Ta-Ag_3_PO_4_ on the growth of bacteria with and without sonication [61]. Three sets of experiment were designed. First set: bacterial cells treated with nanoparticles but without sonication; second set: bacterial cells treated with nanoparticles with sonication i.e., the bacterial cells treated with Ag_3_PO_4_, Se-Ag_3_PO_4_, Ag-Ag_3_PO_4_, and Ta-Ag_3_PO_4_ at their sub-MIC values and third set: bacterial cells without nanoparticles and sonication (negative control). Then, all three sets were incubated for 16 h at 37 °C. After incubation, cells treated with nanoparticles without sonication (first set); cells treated with nanoparticles having 5 min of sonication (second set), and bacterial cells without nanoparticles and sonication (third set) were serially diluted using a tenfold serial dilution method in a 10 mL tube and then 100 μL of diluted bacteria from dilution factor 3 was plated onto nutrient agar plates and then kept overnight at 37 °C in an incubator. Finally, the number of colonies on agar plates was examined by counting the CFU/mL to evaluate the antibacterial potential of the tested materials.

### 3.2. Cytotoxicity of Ag_3_PO_4_, Se-Ag_3_PO_4_, Ag-Ag_3_PO_4_, and Ta-Ag_3_PO_4_

#### 3.2.1. In Vitro Culture and Testing by MTT Method

The cytotoxicity of Ag_3_PO_4,_ Se-Ag_3_PO_4,_ Ag-Ag_3_PO_4_, and Ta-Ag_3_PO_4_ was studied against human colorectal carcinoma cells (HCT-116) and human cervical carcinoma cells (HeLa cells), which were purchased from ATCC, USA. Additionally, as a control, we studied against healthy human embryonic kidney cells (HEK-293) which were purchased from ATCC, USA. The cells culture was maintained in the Dulbecco’s Modified Eagle Medium (DMEM) composed of 10% fetal bovine serum (FBS), penicillin (1%), L-glutamine (5%), streptomycin (1%), and selenium chloride (1%) as reported earlier [62]. The cells were grown in a 5% CO_2_ incubator and an MTT assay was performed according to the previous study [39]. The cells were treated with Ag_3_PO_4,_ Se-Ag_3_PO_4,_ Ag-Ag_3_PO_4_, and Ta-Ag_3_PO_4_ with different concentrations (5–100 µg/mL). Both the control and Ag_3_PO_4,_ Se-Ag_3_PO_4,_ Ag-Ag_3_PO_4,_ Ta-Ag_3_PO_4_ nanoparticles were cured with 10 µL of MTT reagent (5.0 mg/mL) and cells were incubated for 4 more hours. Afterwards, the culture medium was exchanged with DMSO (1%) and absorbance was recorded at 570 nm using an ELISA plate reader to compute % cell viability for statistical analysis. 

#### 3.2.2. Apoptotic Morphology by DAPI Staining

DAPI staining was performed to observe the DNA of cancer cells. Cells were allocated into two groups; the control group where no Ag_3_PO_4,_ Se-Ag_3_PO_4,_ Ag-Ag_3_PO_4_, and Ta-Ag_3_PO_4_ were present, whereas, in the trial group, 40 µg/mL of Ag_3_PO_4,_ Se-Ag_3_PO_4,_ Ag-Ag_3_PO_4,_ Ta-Ag_3_PO_4_ was present. Following 48 h of treatment, ice-cold (4%) paraformaldehyde was introduced to both groups and then triton x-100 in PBS (phosphate buffer saline) was added, followed by treatment with DAPI (1 µg/mL) in the dark and the cells were washed using PBS, cover-slipped and viewed under a confocal scanning microscope. 

## 4. Conclusions

Ag_3_PO_4,_ Se-Ag_3_PO_4,_ Ag-Ag_3_PO_4_, and Ta-Ag_3_PO_4_ nanoparticles were prepared by ultrasonic method and characterized by good pore sizes of less than 5 nm. It was perceived that Ag-Ag_3_PO_4_ exhibited enhanced antibacterial activity followed by Ta-Ag_3_PO_4,_ Ag_3_PO_4_ and Se-Ag_3_PO_4_ against *E. coli*. Whereas the order of antibacterial activity against *S. aureus* was as follows: Ag_3_PO_4_ > Ag-Ag_3_PO_4_ > Ta-Ag_3_PO_4_ > Se-Ag_3_PO_4_, respectively. The antibacterial order almost observes the pore size order with smaller being more effective, except for Se-Ag_3_PO_4_ that was the least effective despite having the smallest pore size. Results indicated that Gram-negative bacteria (*E. coli*) were more susceptible to the tested nanoparticles than Gram-positive bacteria (*S. aureus*). Additionally, the effects of sonication treatment on bacterial growth in the presence of nanoparticles were also examined and it was observed that the viable cell count of bacteria cells was significantly reduced after 5 min of sonication treatment as compared to cells treated without sonication. The IC_50_ values for Ag_3_PO_4,_ Se-Ag_3_PO_4,_ Ag-Ag_3_PO_4_, and Ta-Ag_3_PO_4_ on HCT-116 cells were 39.44, 28.33, 60.24, 58.34 µg/mL; whereas for HeLa cells, they were 65.25, 61.27, 75.52, 72.82 µg/mL, respectively. Furthermore, we found that Ag_3_PO_4,_ Se-Ag_3_PO_4,_ Ag-Ag_3_PO_4_, and Ta-Ag_3_PO_4_ did not have an inhibitory effect on HEK-293 cells, rendering them safe therapeutic candidates without any effects on healthy cells. 

## Figures and Tables

**Table 1:** Zeta potential, particle size, and polydispersity index of synthesized nanoparticles.

Nanoparticles	Zeta Potential (mV)	Particle Size (nm)	Polydispersity Index (PDI)
Ag_3_PO_4_	−40.1 ± 6.63	115	0.509
Se-Ag_3_PO_4_	−5.24 ± 10.8	458	1.00
Ag-Ag_3_PO_4_	−46.6 ± 4.77	426	0.949
Ta-Ag_3_PO_4_	−79.8 ± 7.96	82.78	0.594

**Table 2:** MIC and MBC (mg/mL) values of tested compounds against *E. coli* and *S. aureus*.

	*E. coli*		*S. aureus*	
	MIC	MBC	MIC	MBC
Ag_3_PO_4_	1.0 ± 0.0	2 ± 0.0	2 ± 0.0	4 ± 0.0
Se-Ag_3_PO_4_	8 ± 0.0	16 ± 0.0	4 ± 0.0	8 ± 0.0
Ag-Ag_3_PO_4_	0.125 ± 0.0	0.5 ± 0.0	2 ± 0.0	8 ± 0.0
Ta-Ag_3_PO_4_	0.25 ± 0.0	1 ± 0.0	4 ± 0.0	8 ± 0.0

## Data Availability

Not applicable.

## Supplementary Materials

The following supporting information can be downloaded at: https://www.mdpi.com/article/10.3390/ijms231911403/s1.

## References

[1] Lu Y., Wan X., Li L., Sun P.F., Liu G. (2021). Synthesis of a reusable composite of graphene and silver nanoparticles for catalytic reduction of 4-nitrophenol and performance as anti-colorectal carcinoma. J. Mater. Res. Technol..

[2] Qureshi F., Nawaz M., Rehman S., Almofty S.A., Shahzad S., Nissapatorn V., Taha M. (2020). Synthesis and characterization of cadmium-bismuth microspheres for the catalytic and photocatalytic degradation of organic pollutants, with antibacterial, antioxidant and cytotoxicity assay. J. Photochem. Photobiol. B.

[3] Nawaz M., Akhtar S., Qureshi F., Almofty S.A., Nissapatron V. (2022). Preparation of indium-cadmium sulfide nanoparticles with diverse morphologies: Photocatalytic and cytotoxicity study. J. Mol. Struct..

[4] Abebe B., Murthy H.C.A., Dessie Y. (2020). Synthesis and characterization of Ti–Fe oxide nanomaterials: Adsorption–degradation of methyl orange dye. Arab. J. Sci. Eng..

[5] Liu Y.Y., Guo X., Chen Z., Zhang W., Wang Y., Zheng Y., Tang X., Zhang M., Peng Z., Li R. (2020). Microwave-synthesis of g-C_3_N_4_ nanoribbons assembled seaweed-like architecture with enhanced photocatalytic property. Appl. Catal. B Environ..

[6] Liao S.H., Liu C.H., Bastakoti B.P., Suzuki N., Chang Y., Yamauchi Y., Wu K.C. (2015). Pulmonary protective effects of ultrasonic green synthesis of gold nanoparticles mediated by pectin on Methotrexate-induced acute lung injury in lung BEAS-2B, WI-38, CCD-19Lu, IMR-90, MRC-5, and HEL 299 cell lines. Int. J. Nanomed..

[7] Veisi H., Najafi S., Hemmati S. (2018). Pd(II)/Pd(0) anchored to magnetic nanoparticles (Fe_3_O_4_) modified with biguanidine-chitosan polymer as a novel nanocatalyst for Suzuki-Miyaura coupling reactions. Int. J. Biol. Macromol..

[8] Arunachalam K.D., Annamalai S.K., Hari S. (2003). One-step green synthesis and characterization of leaf extract-mediated biocompatible silver and gold nanoparticles from Memecylon umbellatum. Int. J. Nanomed..

[9] You C., Han C., Wang X., Zheng Y., Li Q., Hu X., Sun H. (2012). The progress of silver nanoparticles in the antibacterial mechanism, clinical application and cytotoxicity. Mol. Biol. Rep..

[10] Mao B.-H., Tsai J.-C., Chen C.-W., Yan S.-J., Wang Y.-J. (2016). Mechanisms of silver nanoparticle-induced toxicity and important role of autophagy. Nanotoxicology.

[11] Mahboob T., Nawaz M., Pereira M.L., Tan T.C., Samudi C., Sekaran S.D., Wiart C., Nissapatorn V. (2020). PLGA nanoparticles loaded with Gallic acid- a constituent of *Leea indica* against *Acanthamoeba triangularis*. Sci. Rep..

[12] Jannat K., Paul A.K., Bondhon T.A., Hasan A., Nawaz M., Jahan R., Mahboob T., Nissapatorn V., Wilairatana P., Pereira M.L. (2021). Nanotechnology applications of flavonoids for viral diseases. Pharmaceutics.

[13] Al-Suhaimi E.A., Firdos A., Khan F.A., Aljafary M.A., Baykal A., Homeida A.M. (2021). Emerging trends in the delivery of nanoformulated oxytocin across Blood-Brain barrier. Int. J. Pharm..

[14] Lim C.L., Raju C.S., Mahboob T., Kayesth S., Gupta K., Jain G.K., Dhobi M., Nawaz M., Wilairatana P., Pereira M.L. (2022). Precision and Advanced Nano-Phytopharmaceuticals for Therapeutic Applications. Nanomaterials.

[15] De Jong W.H., Borm P.J. (2008). Drug delivery and nanoparticles: Applications and hazards. Int. J. Nanomed..

[16] Borm P.J., Robbins D., Haubold S., Kuhlbusch T., Fissan H., Donaldson K., Schins R., Stone V., Kreyling W., Lademann J. (2006). The potential risks of nanomaterials: A review carried out for ECETOC. Part. Fibre Toxicol..

[17] Patra J.K., Das G., Fraceto L.F., Campos E.V.R., Rodriguez-Torres M.D.P., Acosta-Torres L.S., Diaz Torres L.A., Grillo R., Swamy M.K., Sharma S. (2018). Nano based drug delivery systems: Recent developments and future prospects. J. Nanobiotechnol..

[18] Stapleton P.A., Nurkiewicz T.R. (2014). Vascular distribution of nanomaterials. Wiley Interdiscip. Rev. Nanomed. Nanobiotechnol..

[19] Habibi-Yangjeh A., Asadzadeh-Khaneghah S., Feizpoor S., Rouhi A. (2020). Review on heterogeneous photocatalytic disinfection of waterborne, airborne, and foodborne viruses: Can we win against pathogenic viruses?. J. Colloid Interface Sci..

[20] Hong X., Li M., Shan S., Hui K.S., Mo M., Yuan X. (2016). Chloride ion-driven transformation from Ag_3_PO_4_ to AgCl on the hydroxyapatite support and its dual antibacterial effect against *Escherichia coli* under visible light irradiation. Environ. Sci. Pollut. Res. Int..

[21] Pant B., Park M., Park S.-J. (2019). One-step synthesis of silver nanoparticles embedded polyurethane nano-fiber/net structured membrane as an effective antibacterial medium. Polymers.

[22] Shen W., Li P., Feng H., Ge Y., Liu Z., Feng L. (2017). The bactericidal mechanism of action against *Staphylococcus aureus* for AgO nanoparticles. Mater. Sci. Eng. C.

[23] Thiyagarajan S., Singh S., Bahadur D. (2016). Reusable sunlight activated photocatalyst Ag_3_PO_4_ and its significant antibacterial activity. Mater. Chem. Phys..

[24] Xue J., Zan G., Wu Q., Deng B., Zhng Y., Huang H., Zhang X. (2016). Integrated nanotechnology for synergism and degradation of fungicide SOPP using micro/nano-Ag_3_PO_4_. Inorg. Chem. Front..

[25] Panthi G., Ranjit R., Kim H.Y., Mulmi D.D. (2018). Size dependent optical and antibacterial properties of Ag_3_PO_4_ synthesized by facile precipitation and colloidal approach in aqueous solution. Optik.

[26] Wu A., Tian C., Chang W., Hong Y., Zhang Q., Qu Y., Fu H. (2013). Morphology-controlled synthesis of Ag_3_PO_4_ nano/microcrystals and their antibacterial properties. Mater. Res. Bull..

[27] Trench A.B., Machado T.R., Gouveia A.F., Foggi C.C., Teodoro V., Sánchez-Montes I., Teixeira M.M., da Trindade L.G., Jacomaci N., Perrin A. (2020). Rational Design of W-Doped Ag_3_PO_4_ as an Efficient Antibacterial Agent and Photocatalyst for Organic Pollutant Degradation. ACS Omega.

[28] Shao J., Ma J., Lin L., Wang B., Jansen J.A., Walboomers X.F., Zuo Y., Yang F. (2019). Three-dimensional Printing of Drug-loaded Scaffolds for Antibacterial and Analgesic Applications. Tissue Eng. Part C.

[29] Zhang Y., Zhang X., Hu R., Yang Y., Li P., Wu Q. (2019). Bifunctional Nano-Ag3PO4 with Capabilities of Enhancing Ceftazidime for Sterilization and Removing Residues. RSC Adv..

[30] Zhuang J., Liu J., Wu Z., Li Z., Zhu K., Yan K., Xu Y., Huang Y., Lin Z. (2019). Formation of Ag_3_PO_4_/AgBr composites with Z-scheme configuration by an in situ strategy and their superior photocatalytic activity with excellent anti-photocorrosion performance. J. Mater. Sci. Mater. Electron..

[31] Gao R., Song J., Hu Y., Zhang X., Gong S., Li W. (2017). Facile Synthesis of Ag/Ag_3_PO_4_ Composites with Highly Efficient and Stable Photocatalytic Performance under Visible Light. J. Chin. Chem. Soc..

[32] Xiaohong W., Jian J., Zhengqiu Y., Jianxian Z., Lei Z., Taofen W., Hu Z. (2020). In situ loading of polyurethane/negative ion powder composite film with visible-light-responsive Ag_3_PO_4_@AgBr particles for photocatalytic and antibacterial applications. Eur. Polym. J..

[33] Hossein B., Mohammad A.Z., Vahid V. (2020). Antibacterial and antifouling properties of Ag_3_PO_4_/GO nanocomposite blended polyethersulfone membrane applied in dye separation. J. Water Process Eng..

[34] Kaili M., Yao Z., Xiaoying Z., Jian R., Fengxian Q., Huayou C., Jinchao X., Dongya Y., Tao Z. (2020). Effective loading of well-doped ZnO/Ag_3_PO_4_ nanohybrids on magnetic core via one step for promoting its photocatalytic antibacterial activity. Colloids Surf. A.

[35] Qinqing W., Shuting J., Suyun L., Xueqing Z., Junhui Y., Pei L., Wenyan S., Minghong W., Longxiang S. (2021). Electrospinning visible light response Bi_2_MoO_6_/Ag_3_PO_4_ composite photocatalytic nanofibers with enhanced photocatalytic and antibacterial activity. App. Surf. Sci..

[36] Lyu Y., Wei F., Zhang T., Luo L., Pan Y., Yang X., Yu H., Zhou S. (2021). Different antibacterial effect of Ag_3_PO_4_/TiO_2_ heterojunctions and the TiO_2_ polymorphs. J. Alloys Comp..

[37] Wang X., Utsumi M., Yang Y., Li D., Zhao Y., Zhang Z., Feng C., Sugiura N., Cheng J.J. (2015). Degradation of microcystin-LR by highly efficient AgBr/Ag_3_PO_4_/TiO_2_ heterojunction photocatalyst under simulated solar light irradiation. Appl. Surf. Sci..

[38] Liu Y., Fang L., Lu H., Liu L., Wang H., Hu C. (2012). Highly efficient and stable Ag/Ag_3_PO_4_ plasmonic photocatalyst in visible light. Catal. Commun..

[39] Filipović N., Ušjak D., Milenković M.T., Zheng K., Liverani L., Boccaccini A.R., Stevanović M.M. (2021). Comparative study of the antimicrobial activity of selenium nanoparticles with different surface chemistry and structure. Front. Bioeng. Biotechnol..

[40] Geoffrion L.D., Hesabizadeh T., Medina-Cruz D., Kusper M., Taylor P., Vernet-Crua A., Chen J., Ajo A., Webster T.J., Guisbiers G. (2020). Naked selenium nanoparticles for antibacterial and anticancer treatments. ACS Omega.

[41] Harrison P.L., Harrison T., Stockley I., Smith T.J. (2017). Does tantalum exhibit any intrinsic antimicrobial or antibiofilm properties?. Bone Joint J..

[42] Lin L., Wan H., Mia R., Jiang H., Liu H., Mahmud S. (2022). Bioreduction and Stabilization of Antibacterial Nanosilver Using *Radix Lithospermi* Phytonutrients for Azo-contaminated Wastewater Treatment: Synthesis, Optimization and Characterization. J. Clust. Sci..

[43] Wang H., Zhang G., Mia R., Wang W., Xie L., Lü S., Mahmud S., Liu H. (2022). Bioreduction (Ag+ to Ag0) and stabilization of silver nanocatalyst using hyaluronate biopolymer for azo-contaminated wastewater treatment. J. Alloys Compd..

[44] Mia R., Sk S., Oli Z.B.S., Ahmed T., Kabir S., Waqar A. (2021). Functionalizing cotton fabrics through herbally synthesized nanosilver. Cleaner Eng. Technol..

[45] Zhang G., Wan H., Mia R., Huang Q., Liu H., Mahmud S. (2022). Fabrication and stabilization of nanosilver using Houttugniae for antibacterial and catalytic application. Int. J. Environ. Anal. Chem..

[46] Liang Q.H., Shi Y., Ma W.J., Li Z., Yang X.M. (2012). Enhanced photocatalytic activity and structural stability by hybridizing Ag_3_PO_4_ nanospheres with graphene oxide sheets. Phys. Chem. Chem. Phys..

[47] Nawaz M., Mou F., Xu L., Guan J. (2018). Effect of solvents and reaction parameters on the morphology of Ta_2_O_5_ and photocatalytic activity. J. Mol. Liquids.

[48] Babayevska N., Przysiecka L., Iatsunskyi I., Nowaczyk G., Jarek M., Janiszewska E., Jurga S. (2022). ZnO size and shape effect on antibacterial activity and cytotoxicity profile. Sci. Rep..

[49] Yamamoto O. (2013). Influence of particle size on the antibacterial activity of zinc oxide. Int. J. Inorg. Mater..

[50] Baig U., Gondal M.A., Ansari M.A., Dastageer M.A., Sajid M., Falath W.S. (2021). Rapid synthesis and characterization of advanced ceramic-polymeric nanocomposites for efficient photocatalytic decontamination of hazardous organic pollutant under visible light and inhibition of microbial biofilm. Ceram. Int..

[51] González S.C.E., Bolaina-Lorenzo E., Pérez-Trujillo J.J., Puente-Urbina B.A., Rodríguez-Fernández O., Fonseca-García A., Betancourt-Galindo R. (2021). Antibacterial and anticancer activity of ZnO with different morphologies: A comparative study. 3 Biotech.

[52] Song C., Labhasetwar V., Cui X., Underwood T., Levy R.J. (1998). Arterial uptake of biodegradable nanoparticles for intravascular local drug delivery: Results with an acute dog model. J. Control. Release.

[53] Barua S., Mitragotri S. (2014). Challenges associated with Penetration of Nanoparticles across Cell and Tissue Barriers: A Review of Current Status and Future Prospects. Nano Today.

[54] Contini C., Hindley J.W., Macdonald T.J., Barritt J.D., Ces O., Quirke N. (2020). Size dependency of gold nanoparticles interacting with model membranes. Commun. Chem..

[55] Nawaz M., Almofty S.A., Qureshi F. (2018). Preparation, formation mechanism, photocatalytic, cytotoxicity and antioxidant activity of sodium niobate nanocubes. PLoS ONE.

[56] El Rayes S.M., Aboelmagd A., Gomaa M.S., Ali I.A.I., Fathalla W., Pottoo F., Khan F.A. (2019). Convenient synthesis and anticancer activity of methyl 2-[3-(3-Phenyl-quinoxalin-2-ylsulfanyl)propanamido]alkanoates and N-Alkyl 3-((3-Phenyl-quinoxalin-2-yl)sulfanyl) propanamides. ACS Omega.

[57] Khan F.A., Lammari N., Muhammad S.A.S., Alkhater K.M., Asiri S., Akhtar S., Almansour I., Alamoudi W., Haroun W., Louaer W. (2020). Quantum dots encapsulated with curcumin inhibit the growth of colon cancer, breast cancer and bacterial cells. Nanomedicine.

[58] Almofty S.A., Nawaz M., Qureshi F., Al-Mutairi R. (2022). Hydrothermal Synthesis of β-Nb_2_ZnO_6_ Nanoparticles for Photocatalytic Degradation of Methyl Orange and Cytotoxicity Study. Int. J. Mol. Sci..

[59] Nawaz M., Ansari M.A., Pérez Paz A., Hisaindee S., Qureshi F., Ul-Hamid A., Hakeem A., Taha M. (2022). Sonochemical synthesis of ZnCo_2_O_4_/Ag_3_PO_4_ heterojunction photocatalysts for the degradation of organic pollutants and pathogens: A combined experimental and computational study. New J. Chem..

[60] Ansari M.A., Kalam A., Al-Sehemi A.G., Alomary M.N., AlYahya S., Aziz M.K., Srivastava S., Alghamdi S., Akhtar S., Almalki H.D. (2021). Counteraction of biofilm formation and antimicrobial potential of *Terminalia catappa* functionalized silver nanoparticles against *Candida albicans* and multidrug-resistant gram-negative and gram-positive bacteria. Antibiotics.

[61] Ansari M.A., Akhtar S., Rauf M.A., Alomary M.N., AlYahya S., Alghamdi S., Almessiere M.A., Baykal A., Khan F., Adil S.F. (2021). Sol-gel synthesis of dy-substituted Ni0. 4Cu0. 2Zn0. 4 (Fe2-xDyx) O4 nano spinel ferrites and evaluation of their antibacterial, antifungal, antibiofilm and anticancer potentialities for biomedical application. Int. J. Nanomed..

[62] Khan F.A., Akhtar S., Almohazey D., Alomari M., Almofty S.A., Eliassari A. (2018). Fluorescent magnetic submicronic polymer (FMSP) nanoparticles induce cell death in human colorectal carcinoma cells. Artif. Cells Nanomed. Biotechnol..

