# Supplementary material for: Synthesis of M-Ag3PO4, (M = Se, Ag, Ta) Nanoparticles and Their Antibacterial and Cytotoxicity Study
Source: Int J Mol Sci. 2022 Sep 27;23(19):11403. doi: 10.3390/ijms231911403 (PMC9569642; doi:10.3390/ijms231911403)
Supplement: Supplementary file 1 [file ijms-23-11403-s001.zip › ijms-1886450-supplementary.pdf]

Synthesis of M-Ag_3PO_4, (M= Se, Ag, Ta) Nanoparticles and their Antibacterial and Cytotoxicity Study

Faiza Qureshi[1,2], Muhammad Nawaz[2*], Mohammad Azam Ansari[3], Firdos Alam Khan[4], Mahmoud M. Berekaa[5], Samar A. Abubshait[2,6], Rayyanah Al-Mutairi[2], Alok K. Paul[7], Veeranoot Nissapatorn[8], Maria de Lourdes Pereira[9*], and Polrat Wilairatana[10]

1 Deanship of Scientific Research, Imam Abdulrahman Bin Faisal University, P.0. Box 1982, Dammam 31441, Saudi Arabia
2 Department of Nano-Medicine Research, Institute for Research and Medical Consultations (IRMC), Imam Abdulrahman Bin Faisal University, P.0. Box 1982, Dammam 31441, Saudi Arabia
3 Department of Epidemic Disease Research, Institutes for Research and Medical Consultations (IRMC), Imam Abdulrahman Bin Faisal University, P.O. Box 1982, Dammam 31441, Saudi Arabia
4 Department of Stem Cell Research, Institute for Research and Medical Consultations (IRMC), Imam Abdulrahman Bin Faisal University, P.0. Box 1982, Dammam 31441, Saudi Arabia
5 Environmental Health Department, College of Public Health, Imam Abdulrahman Bin Faisal University, P.0. Box 1982, Dammam 31441, Saudi Arabia
6 Department of Chemistry, College of Science and Basic & Applied Scientific Research Centre, Imam Abdulrahman Bin Faisal University, P.0. Box 1982, Dammam 31441, Saudi Arabia
7 School of Pharmacy and Pharmacology, University of Tasmania, Hobart, TAS 7001, Australia
8 School of Allied Health Sciences, World Union for Herbal Drug Discovery (WUHeDD), and Research Excellence Center for Innovation and Health Products (RECIHP), Walailak University, Nakhon Si Thammarat 80160, Thailand
9 CICECO-Aveiro Institute of Materials & Department of Medical Sciences,University of Aveiro, 3810-193 Aveiro, Portugal
10 Department of Clinical Tropical Medicine, Faculty of Tropical Medicine, Mahidol University, Bangkok 10400, Thailand
* Correspondence: mnnmuhammad@iau.edu.sa (M.N.); mlourdespereira@ua.pt (M.d.L.P.)

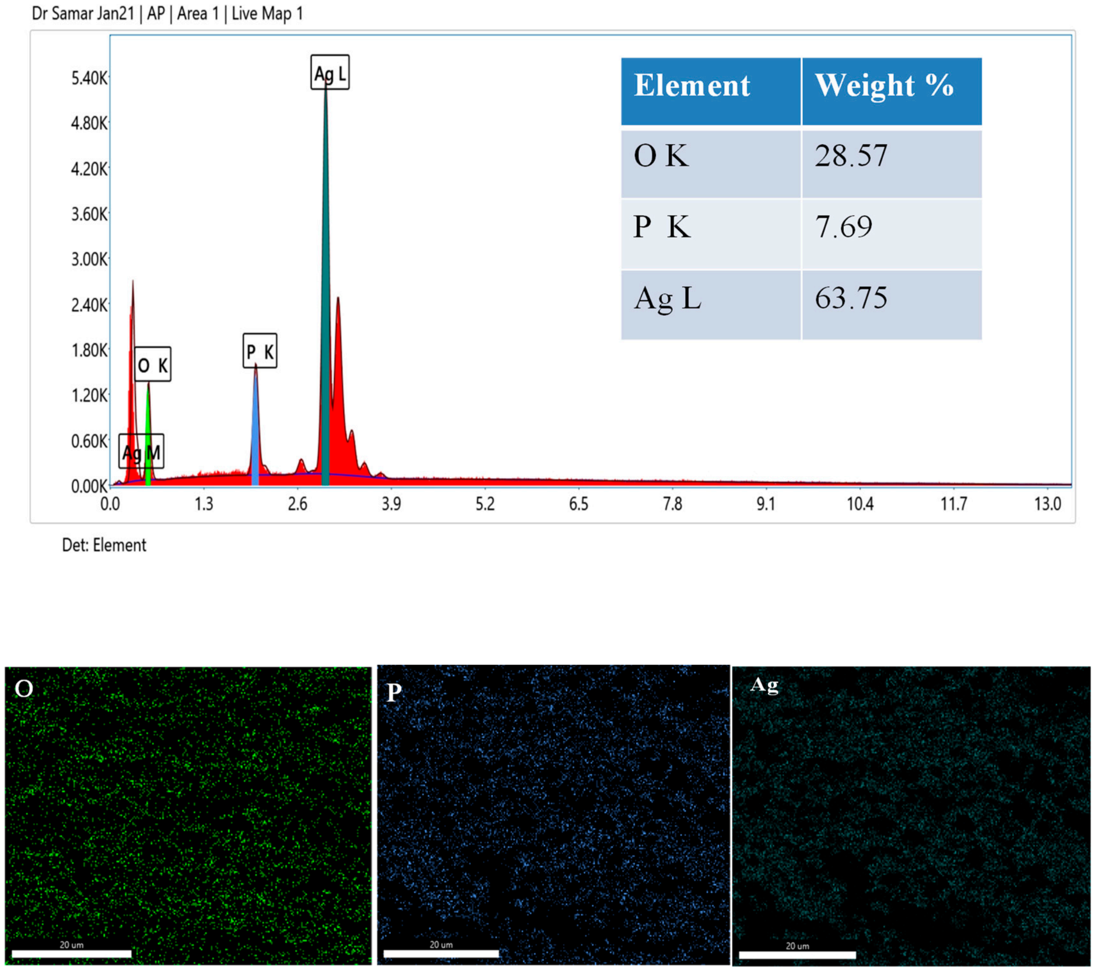

Figure S1. EDX spectrum and elemental (O, P, Ag) mapping of Ag_3PO_4 nanoparticles.

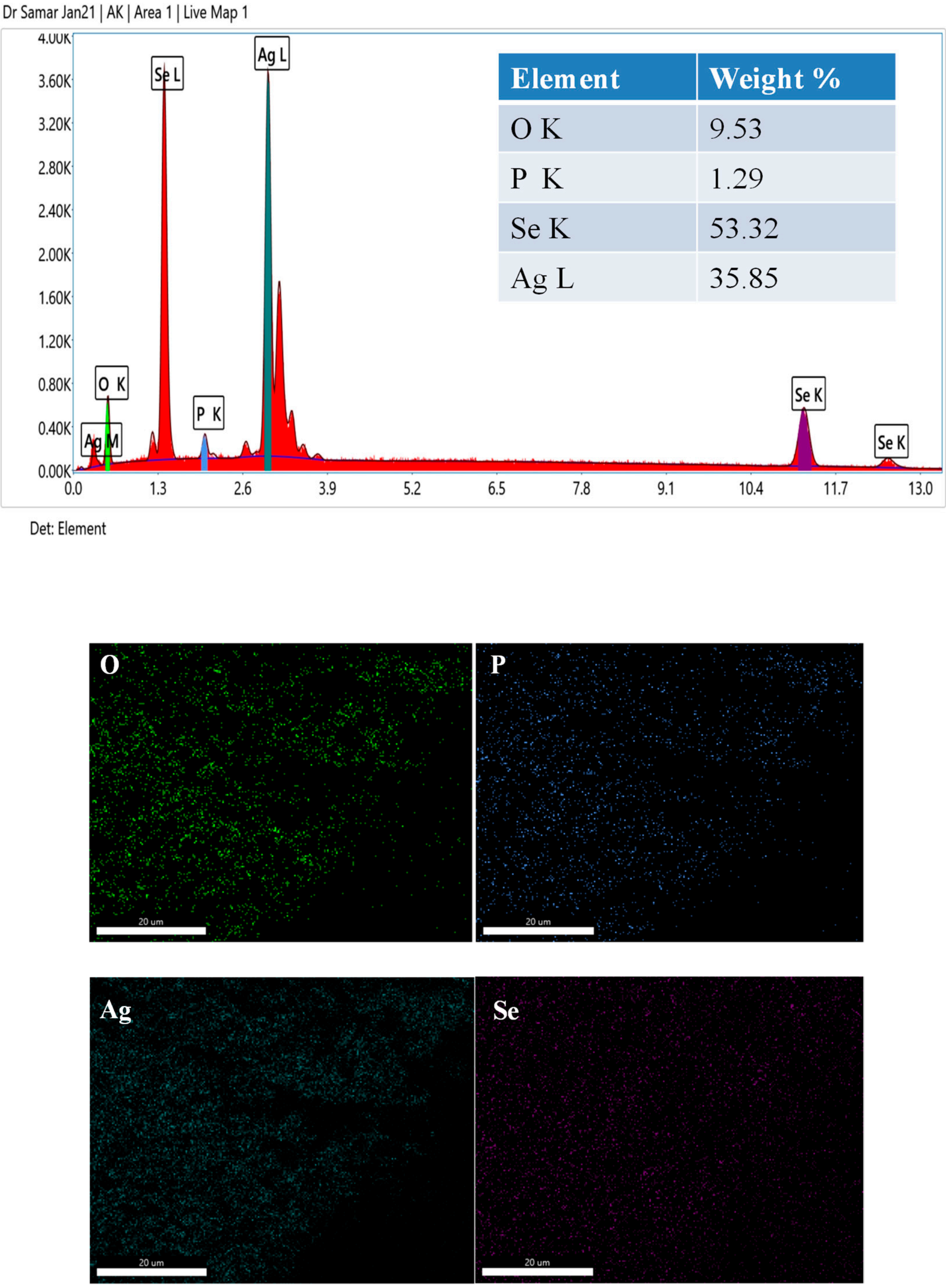

Figure S2. EDX spectrum and elemental (O, P, Ag, Se) mapping of Se-Ag_3PO_4 nanoparticles.

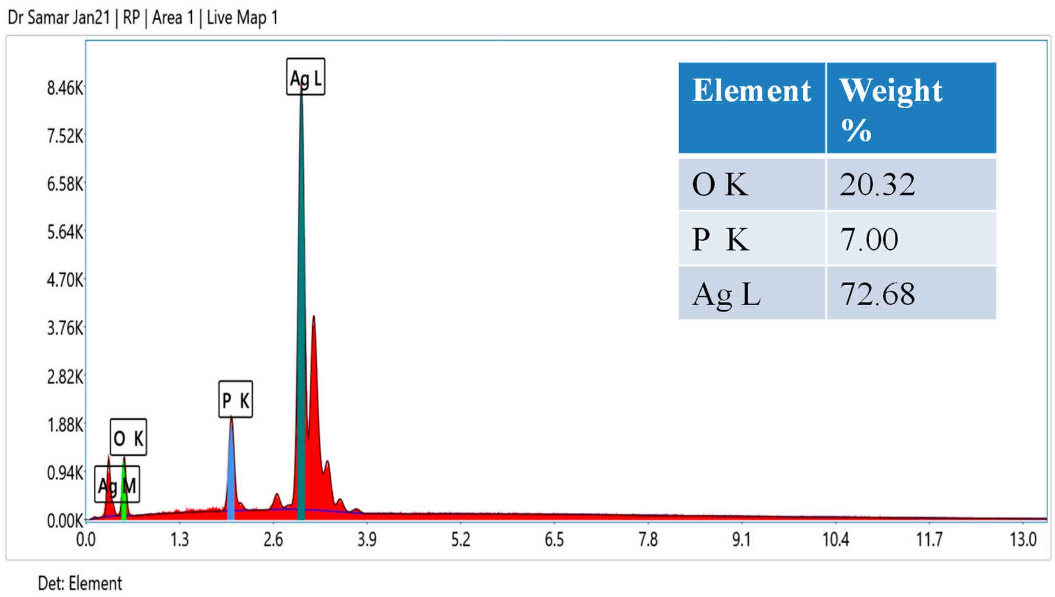

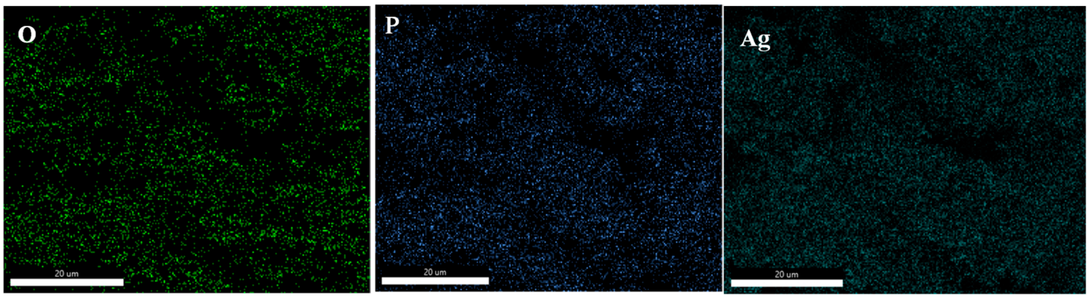

Figure S3. EDX spectrum and elemental (O, P, Ag) mapping of Ag-Ag_3PO_4 nanoparticles.

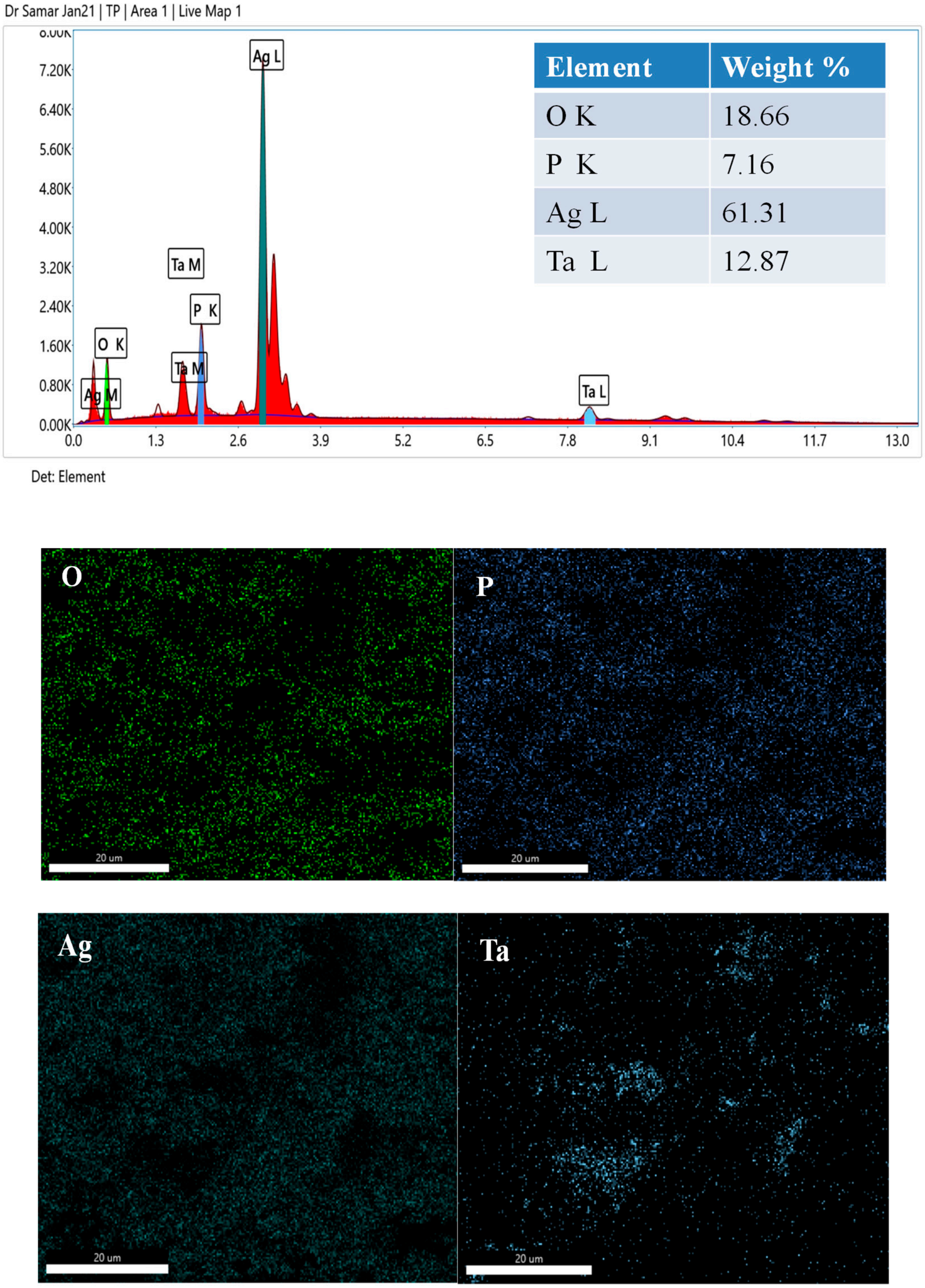

Figure S4. EDX spectrum and elemental (O, P, Ag, Ta) mapping of Ta-Ag_3PO_4 nanoparticles.

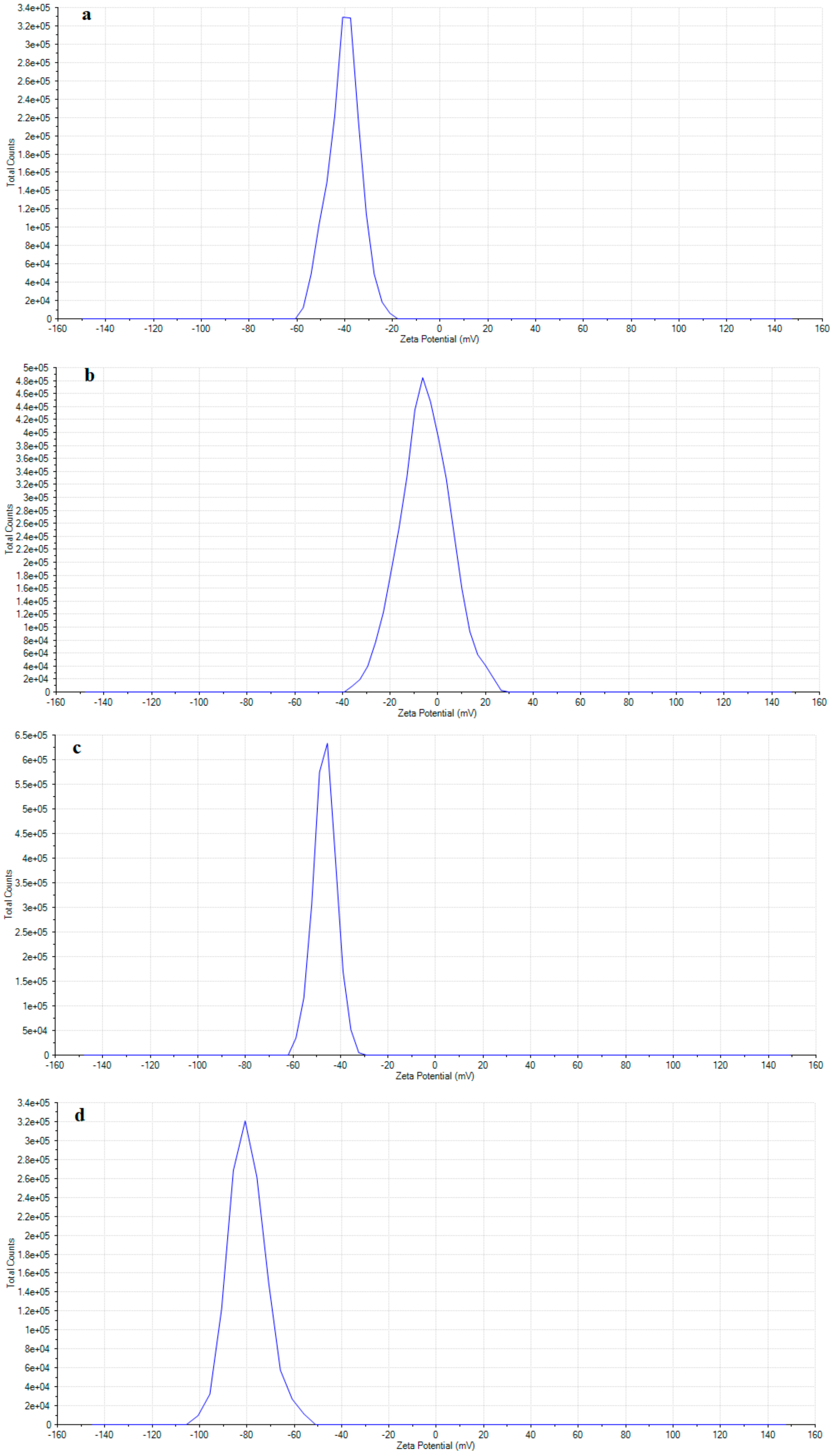

Figure S5. Zeta potential of Ag_3PO_4 (**a**), Se-Ag_3PO_4 (**b**), Ag-Ag_3PO_4 (**c**), Ta-Ag_3PO_4 (**d**) nanoparticles.

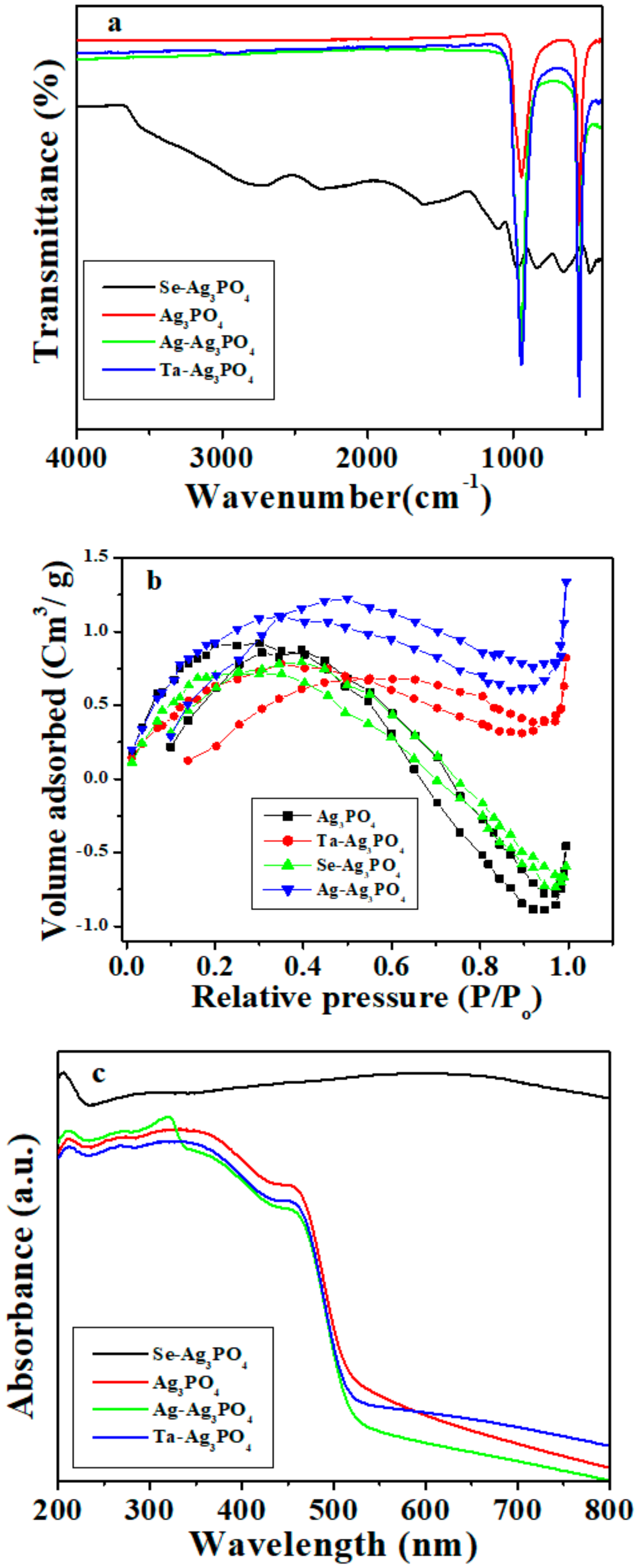

Figure S6. FTIR spectra (**a**), N_2-adsorption-desorption isotherms (**b**) and UV-Visible spectra (**c**) of Ag_3PO_4 , Se-Ag_3PO_4, Ag-Ag_3PO_4, and Ta-Ag_3PO_4 nanoparticles.
